# Chronic Caffeine Consumption Prevents Body Weight Gain and Glucose Intolerance in High-Fat Diet-Induced Obesity Mice Model

**DOI:** 10.3390/metabo16060381

**Published:** 2026-05-31

**Authors:** Giuseppe Faraco, Natália F. Mendes, Luisa O. Schmitt, Tamires S. Stivanin, Elisa Gaspar, Nicolle Platt, Manuella P. Kaster, Joana M. Gaspar

**Affiliations:** 1 Laboratory of Neuroimmune-Metabolism, Federal University of Santa Catarina, Florianopolis 88040-900, Brazil; 2 School of Medical Sciences, Department of Translational Medicine (Section of Pharmacology), University of Campinas, Campinas 13083-887, Brazil; natocrc@unicamp.br; 3 Graduate Program in Biochemistry, Federal University of Santa Catarina, Florianopolis 88040-900, Brazil; 4 Laboratory of Translational Neuroscience, Department of Biochemistry, Federal University of Santa Catarina (UFSC), Florianopolis 88040-970, Brazil

**Keywords:** obesity, caffeine, food intake, hypothalamic neuropeptides

## Abstract

**Highlights:**

**What are the main findings?**

- Caffeine reduces body weight gain in HFD animals.
- Caffeine reduces adiposity accumulation in HFD animals.
- Caffeine improves glucose tolerance in HFD animals.
- Caffeine reduces feed efficiency despite the increase in food intake in HFD animals.

**What are the implications of the main findings?**

- Caffeine has a beneficial effect on energy homeostasis.
- Caffeine has a beneficial effect on metabolic health.

**Abstract:**

**Background/Objectives**: Caffeine consumption has been reported to have beneficial effects in metabolic disorders; however, its effects on food intake are not fully elucidated. This study evaluated the impact of chronic caffeine consumption on weight gain, food intake, and metabolic parameters in C57BL/6 male mice. **Methods**: Eight-week-old male mice (28 animals) were divided into four groups: control (chow diet), caffeine (chow diet + 1 g/L caffeine in drinking water), high-fat diet (HFD), and HFD + caffeine (HFD + 1 g/L caffeine in drinking water). Diets and caffeine were provided ad libitum for 8 weeks. Food and water intake were recorded weekly, and blood glucose was measured every 4 weeks. After 8 weeks of diet and caffeine exposure, metabolic tests were conducted, and tissues were collected for biochemical analysis. **Results**: HFD consumption for 8 weeks induced an increase in body weight and adiposity compared to the chow diet, without changes in food intake. Caffeine consumption prevented body weight gain and adiposity, although it increased food intake. Caffeine also improved glucose tolerance in the HFD mouse model, without changes in random blood glucose, triglyceride, or cholesterol levels. Analysis of hypothalamic neuropeptide (Agrp, NPY, Pomc, Cart), involved in the control of food intake, showed no differences in expression. There were also no changes observed in locomotion nor in anxiety-like behavior. **Conclusions**: In conclusion, chronic high-fat diet (HFD) exposure induced obesity characterized by increased body weight and adiposity without altering food intake. Chronic caffeine consumption counteracted HFD-induced weight gain and fat accumulation and improved glucose tolerance, despite increasing food intake. Importantly, caffeine consumption in the HFD group did not affect locomotor activity or anxiety-like behavior, suggesting that its metabolic effects are not driven by changes in general activity or emotional state. Overall, these findings indicate that chronic caffeine consumption improves metabolic homeostasis in HFD-fed mice.

## 1. Introduction

Obesity is a global epidemic disease. It was estimated in 2021 that around 1 billion adult males and 1.11 billion adult females were overweight and obese, corresponding to 45% of the adult population. It was predicted that by 2050, almost two in three adults over the age of 25 will be overweight or obese [1].

Obesity has been defined lately as a condition characterized by excessive accumulation of fat, with or without abnormal function of the adipose tissue and other organs. Obesity results from a dysregulation of energy homeostasis mechanisms. More recently, the definition of obesity changed with a differentiation in clinical and preclinical obesity, which is related to the presence or absence of tissue dysfunction and comorbidities [2]. Obesity is a risk factor for several metabolic and cardiovascular diseases as well as for psychiatric disorders. In 2021, 3.71 million deaths and 129 million disability-adjusted life years (DALYs) were attributable to being overweight and obesity [3].

A multifaceted interaction among diverse factors contributes to obesity, including genetic, epigenetic, behavioral, socioeconomic, cultural, and environmental factors [2,4,5]. Together, these factors converge to central and peripheral impairment of energy homeostasis (that might even occur at early stages), disrupting the signals for energy intake and expenditure, which leads to pathognomonic excessive fat accumulation and the metabolic dysfunction of obesity [5].

The hypothalamus is the central regulator of energy homeostasis, controlling food intake, satiety, and energy expenditure. The hypothalamus receives peripheral input from multiple sources that are integrated primarily into the arcuate nucleus, which contains two critical neuronal populations. The orexigenic neurons, specifically AgRP/NPY neurons, promote food intake, and the anorexigenic POMC/Cart neurons suppress appetite (increase satiety) and increase energy expenditure. The balance between these two populations controls our feeding behavior and metabolic rate. The hypothalamus also plays a crucial role in the central regulation of peripheral carbohydrates and lipid metabolism [6].

Caffeine, the most widely consumed psychoactive substance in the world, acts at physiologically relevant doses as a non-selective antagonist of adenosine receptors, especially A1R and A2AR [7,8], which are expressed in neurons and glial cells across brain regions regulating cognition and emotional behavior [9,10]. Caffeine is mostly used to promote wakefulness and enhance alertness by enhancing dopaminergic (DA) signaling in the brain, particularly within reward-related circuits such as the mesolimbic pathway and nucleus accumbens [11,12]. Through its actions on central adenosine, dopamine, and glutamate systems, caffeine produces both stimulating and rewarding effects that can foster tolerance and habitual use. Recently it was demonstrated that coffee drinkers exhibited higher impulsivity and emotional reactivity compared to non-coffee drinkers [13]. Dopamine has been proposed to mediate some of the behavioral effects of caffeine [12,14]. Enhanced dopaminergic signaling within reward-related regions, including the nucleus accumbens and mesolimbic pathways, may alter the hedonic aspects of feeding behavior by influencing motivation and food reward perception [15]. Dopamine signaling orchestrates multiple behavioral processes, including motivation, reward valuation, and reinforcement learning, providing a critical neural signal for adaptive behaviors [12,16,17].In the context of feeding behavior, dopamine plays a central role in regulating the motivational and hedonic aspects of food intake rather than directly controlling homeostatic hunger mechanisms. Dopaminergic pathways, particularly projections from the ventral tegmental area to the nucleus accumbens and prefrontal cortex, are involved in encoding food reward, incentive salience, and the motivational drive to seek and consume food. Alterations in dopamine signaling have been associated with changes in feeding behavior, overeating, and obesity, highlighting the importance of dopaminergic neurotransmission in integrating reward processing with energy balance and food-related decision making. Because dopaminergic signaling contributes significantly to reward-driven feeding behavior, caffeine-mediated modulation of dopamine neurotransmission may influence not only homeostatic feeding but also the consumption of highly palatable foods [18,19,20]. Furthermore, dopamine receptor modulation by caffeine can play an important role in controlling glucose homeostasis, as emerging evidence suggests that the central dopaminergic system has intrinsic crosstalk with hepatic aminotransferase activity, key enzymes in the regulation of the shift in energetic pathways [21,22,23].

Previous studies have shown that caffeine consumption is associated with reduced body weight and increased energy, enhancing exercise performance. Caffeine promotes fat oxidation and stimulates thermogenesis [24,25,26,27]. It was found, in a large prospective cohort, that caffeine has an inverse association with the risk of new-onset cardiometabolic multimorbidity [28]. In rats, caffeine presented protective metabolic effects against obesity-associated alterations, including hypometabolism, dyslipidemia, systemic and tissue inflammation, and insulin resistance [29]. Adenosine receptors, particularly A1R, are expressed in orexigenic neurons in the hypothalamus, playing a role in the regulation of energy homeostasis through the regulation of AgRP/NPY gene expression [30]. In mice, acute caffeine administration has been shown to exert anorexigenic effects through A1R, modulating the activity of oxytocin neurons [31]. Recently, it was demonstrated that treatment with caffeine improved body mass index but does not affect the hypothalamic neurotransmitters (GABA, glutamate, glycine and aspartate) in obese rats [32]. However, little is known about the chronic effects caffeine ingestion has on food intake and how this contributes to body weight. In this study, we aimed to analyze the effects of chronic caffeine consumption on body weight gain, food intake, and glucose intolerance in a mouse model of diet-induced obesity.

## 2. Materials and Methods

### 2.1. Animals

Male C57/BL6J mice at 8 weeks of age were acquired and maintained at the Animal Facility of the Department of Biochemistry, Federal University of Santa Catarina (UFSC), and all experimental procedures were conducted in accordance with national and international guidelines for the care and use of laboratory animals. The experimental procedures were approved by the Comissão de Ética no Uso de Animais (CEUA) of the Federal University of Santa Catarina (CEUA number: 1671200524). All animals were group-housed (3 or 4 per cage) in standard cages (41 × 34 cm = 1394 cm^2^ total floor area), with environmental enrichments, and maintained in a 12 h/12 h of light/dark cycle at a temperature of 23 ± 1 °C with controlled humidity, with food and water offered ad libitum.

**Figure 1:**
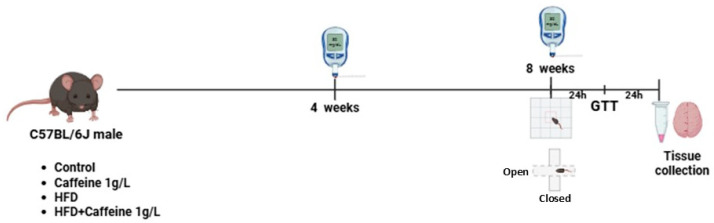
Schematic overview of the experimental design. Male C57BL/6J mice were assigned to one of four groups: control, caffeine (1 g/L in drinking water), high-fat diet (HFD), or HFD supplemented with caffeine (HFD + caffeine). Body weight and fasting blood glucose were monitored at 4 and 8 weeks. At the end of week 8, mice underwent an open-field test and an elevated plus maze test, followed by a glucose tolerance test (GTT) after a 24 h fast. Tissues were collected 24 h after the GTT. Figure created by BioRender.

A total of 28 male C57BL6J mice (assigned with a temporary ID number and divided into different cages) were randomly divided into 4 experimental groups: control group (fed a normal chow diet with 4% of fat); caffeine group (fed a normal chow diet and 1 g/L caffeine (C0750, Sigma Aldrich, St. Louis, MO, USA) in the drinking water); HFD (fed a high-fat diet with 35% fat derived from lard); and HFD + caffeine group (fed HFD diet and 1 g/L caffeine in the drinking water). For both diets, caffeine and water were given ad libitum for 8 weeks. Body weight and food and water intake were measured weekly. Also, every four weeks, a caudal puncture was performed to assess random blood glucose with a glucometer (G-Tech). During the metabolic and behavioral experiments, groups were randomized to account for time-related variation, ensuring that no single group was consistently tested first. The experimental design of this study is shown in Figure 1.

### 2.2. Behavioral Tests

Behavioral experiments were performed after 8 weeks under each diet and caffeine consumption level and were conducted between 9:00 a.m. and 3:00 p.m. (light phase). To evaluate the locomotor and anxiety-like behavior, the open-field test was performed, as previously described [33,34]. Briefly, the apparatus consists of an acrylic box (42 cm^3^), and it is assumed that anxious animals spend more time in the corners of the arena. Animals were placed in the right lower end of the apparatus and were allowed to explore freely for 6 min. The total distance travelled and average speed were used as locomotor parameters, while the number of entries and time spent in the central zone (21 cm^2^) were evaluated as anxiety-related measures.

The elevated plus maze was used to evaluate anxiety-like behavior, and it was performed as previously described [34]. The apparatus consisted of two open arms (1 cm high) and two closed arms (15 cm high) of the same size (30 cm), arranged in a plus shape and connected by a central platform (10 cm^2^). Animals were placed in the center of the maze facing an open arm and were allowed to explore freely for 5 min. The number of entries and time spent in each arm (defined by all four paws entering an arm) were recorded. The test is based on rodents’ natural aversion to open, elevated areas and preference for enclosed spaces, creating a conflict between exploration and anxiety-related avoidance. Reduced exploration of the open arms is interpreted as increased anxiety-like behavior.

### 2.3. Glucose Tolerance Test

Glucose tolerance test (GTT) was performed after eight weeks under each diet and caffeine consumption level to evaluate glucose intolerance. For GTT, mice were fasted for 6 h (8 a.m. until 2 p.m.), and after that a caudal puncture was performed to measure fasting glycemia (t = 0). An intraperitoneal injection of a glucose solution (2 g/kg body mass) was administered, and glycemia was measured after 15, 20, 30, 60, 90 and 120 min. Glycemia was measured using a glucometer (G-Tech, Mountain View, CA, USA). The area under the curve for glucose levels was calculated using the trapezoidal rule for GTT data.

### 2.4. Blood Parameters

Mice were anesthetized with xylazine (10 mg/kg) and ketamine (100 mg/kg) administered intraperitoneally (i.p.) and subsequently euthanized by decapitation a day after the GTT test. All the samples were collected between 9:00 a.m. and 12 p.m. (light phase). Blood was collected by cardiac puncture immediately before euthanasia and centrifuged to obtain serum. Serum cholesterol and triglyceride levels were determined using a commercial kit (Bioclin, Belo Horizonte, Brazil) following the manufacturer’s instructions.

### 2.5. Hypothalamus Neuropeptide Transcriptional Profiling

Hypothalamus samples were collected and stored at −80 °C until use. Total RNA was extracted using the TRI Reagent (Sigma Aldrich) according to the manufacturer’s recommendations. cDNA synthesis was performed using 2.0 μg of total RNA, according to the manufacturer’s recommendations (High-Capacity cDNA Reverse Transcription Kit, Life Technologies, Carlsbad, CA, USA). Each PCR contained 25 ng of reverse-transcribed RNA, 0.25 μL of each specific primer (Applied Biosystems or Integrated DNA Technologies, Waltham or San Diego, CA, USA), Taqman Universal master mix (Life Technologies, Carlsbad, CA, USA) and RNAse-free water, totalizing 10 μL. The analysis of the gene expression was performed in an ABI Prism 7500 sequence detection system (Applied Biosystems, Waltham, CA, USA). GAPDH was used to normalize the relative gene expression in all samples. The primers are listed in Table 1.

### 2.6. Statistical Analysis

Results are presented as individual values, mean ± SEM. Outliers were identified using the ROUT method (robust regression and outlier removal) with Q = 1%. Normality was accessed using Shapiro–Wilk test. Statistical comparisons between different groups were performed using two-way analysis of variance (2-ANOVA) followed by the Sidak multi-comparison test when appropriate, using GraphPad 8 software. Differences were considered significant for *p* ≤ 0.05.

## 3. Results

### 3.1. Body Weight and Adiposity

**Figure 2:**
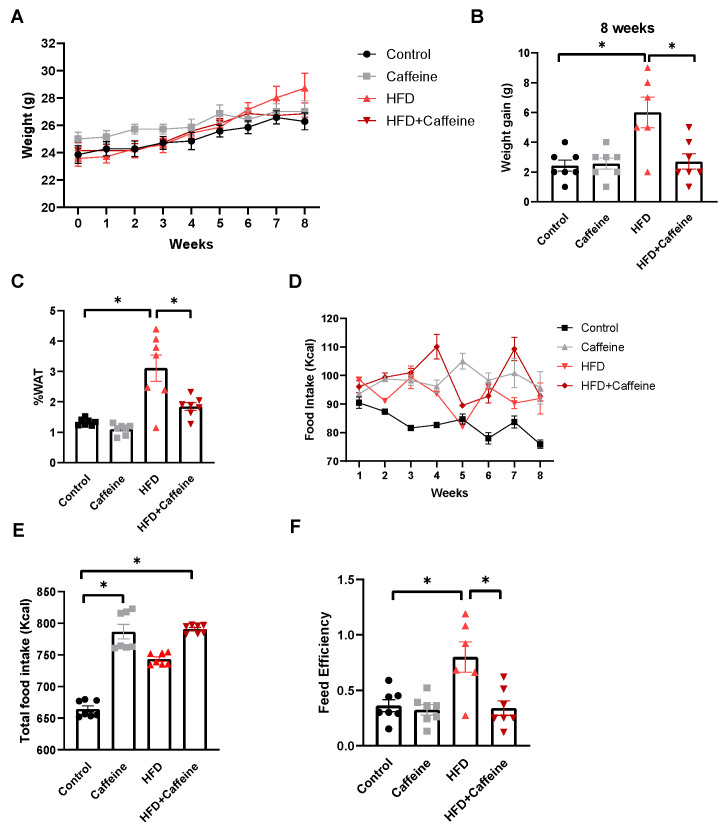
Chronic caffeine consumption prevents body weight gain in HFD mice, despite the increase in food intake. (**A**) Body weight (g) measured weekly over 8 weeks. (**B**) Total weight gain (g) at 8 weeks. (**C**) White adipose tissue mass expressed as a percentage of body weight (%WAT). (**D**) Weekly caloric food intake (kcal). (**E**) Total cumulative food intake (kcal) over the 8-week period. (**F**) Feed efficiency, calculated as weight gain divided by total caloric intake. Data are presented as individual values, mean ± SEM of *n* = 6–7 animals/group; * *p* < 0.05.

Body weight was measured weekly throughout the 8-week experimental period (Figure 2A).

After 8 weeks, the HFD group exhibited higher weight gain compared with the control group (5.96 ± 0.78 g vs. 2.43 ± 0.77 g; *p* = 0.0045; ηp2 diet = 0.61; Figure 2B). Consumption of caffeine reduced weight gain compared with HFD alone (2.71 ± 0.78 g vs. 5.96 ± 0.78 g; *p* = 0.0091; ηp2 treatment = 0.52). A large diet × treatment interaction effect was observed (ηp2 = 0.73), suggesting that the impact of caffeine was particularly pronounced in the diet-induced obesity context. Caffeine alone did not affect weight gain relative to the control group (Figure 2A,B).

Regarding the effect of chronic caffeine consumption, we observed a significant increase in total food intake (kcal) over the 8-week period in the groups receiving caffeine (Figure 2D,E). This effect was characterized by a large magnitude of change (ε2 = 0.86). Post hoc comparisons showed that caffeine increased caloric intake in both the HFD + caffeine (*p* = 0.0468) and caffeine (*p* = 0.0004) groups compared to their respective controls (Figure 2E). A significant interaction between group and time was observed (F(21,192) = 4.954, *p* < 0.0001), indicating that the temporal pattern of caloric intake differed among experimental groups. Significant main effects of time (F(7192) = 4.409, *p* = 0.0001) and group (F(3192) = 67.63, *p* < 0.0001) were also detected (Figure 2D).

However, when feed efficiency (weight gain per unit of feed intake) was measured, two-way ANOVA revealed significant main effects for diet (ηp2 = 0.57) and treatment (ηp2 = 0.62), with a strong interaction between factors (ηp2 = 0.70). Feed efficiency was significantly increased in the HFD group compared with the control group (0.79 ± 0.10 vs. 0.36 ± 0.10; *p* = 0.0079; Figure 2F). Notably, this parameter was significantly decreased in the HFD + caffeine group compared with the HFD group (0.33 ± 0.11 vs. 0.79 ± 0.10; *p* = 0.0054), restoring it to levels similar to the control group, and it was significantly decreased in the HFD + caffeine group compared with the HFD group (Figure 2F).

The percentage of epididymal white adipose tissue was increased in the HFD group compared with the control group (3.11 ± 0.26% vs. 1.34 ± 0.26%; *p* = 0.0030; ηp2 diet = 0.80; Figure 2D). The HFD + caffeine group showed a significant decrease in the percentage of epididymal white adipose tissue compared to the HFD group (1.84 ± 0.26% vs. 3.11 ± 0.26%; *p* = 0.0168; ηp2 treatment = 0.76). An interaction between diet and treatment was observed (ηp2 interaction = 0.56).

### 3.2. Glucose Homeostasis and Lipid Profile

**Figure 3:**
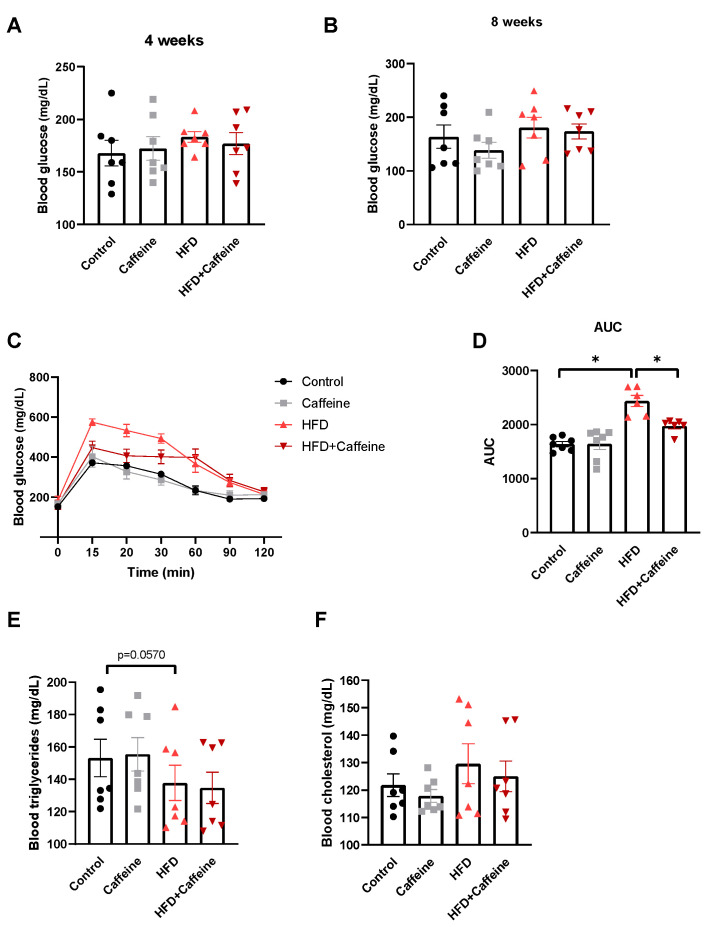
Chronic caffeine consumption improves glucose tolerance in HFD mice. (**A**) Random blood glucose (mg/dL) at 4 weeks. (**B**) Random blood glucose (mg/dL) at 8 weeks. (**C**) Blood glucose levels (mg/dL) during the glucose tolerance test (GTT) at time points 0, 15, 20, 30, 60, 90, and 120 min following intraperitoneal glucose injection. (**D**) Area under the curve (AUC) of the GTT. (**E**) Fasting blood triglyceride levels (mg/dL). (**F**) Fasting blood cholesterol levels (mg/dL). Data are presented as individual values, mean ± SEM of *n* = 7 animals/group. * *p* < 0.05.

Random blood glucose levels at 4 and 8 weeks were comparable between groups (Figure 3A,B). In contrast, the glucose tolerance test revealed an impairment in glucose homeostasis in the HFD group, as shown by an increased area under the curve (AUC) compared with the control group (2433 ± 97 vs. 1639 ± 97; *p* = 0.0073; Figure 3D). Two-way ANOVA showed significant effects for diet (ηp2 = 0.91) and treatment (ηp2 = 0.51), with a strong interaction between both factors (ηp2 = 0.76). Caffeine consumption significantly reduced the AUC in HFD-fed animals compared with the HFD-alone group (1947 ± 121 vs. 2433 ± 97; *p* = 0.0152), indicating an improvement in glucose tolerance.

We also analyzed the serum lipid profile by measuring triglyceride and cholesterol levels in serum (Figure 3E,F). It was observed that diet had a significant main effect on triglyceride levels (ηp2 = 0.84; Figure 3E), although no significant effect of treatment or interaction was observed. Moreover, a trend toward decreased triglyceride levels in the HFD group compared with the control group was found (137.7 ± 10.93 mg/dL vs. 153.2 ± 10.93 mg/dL; *p* = 0.0570). However, caffeine consumption did not significantly alter triglyceride levels in HFD-fed animals. Similarly, no significant differences were found among groups regarding total cholesterol levels (Figure 3F).

### 3.3. Behavioral Assessment

**Figure 4:**
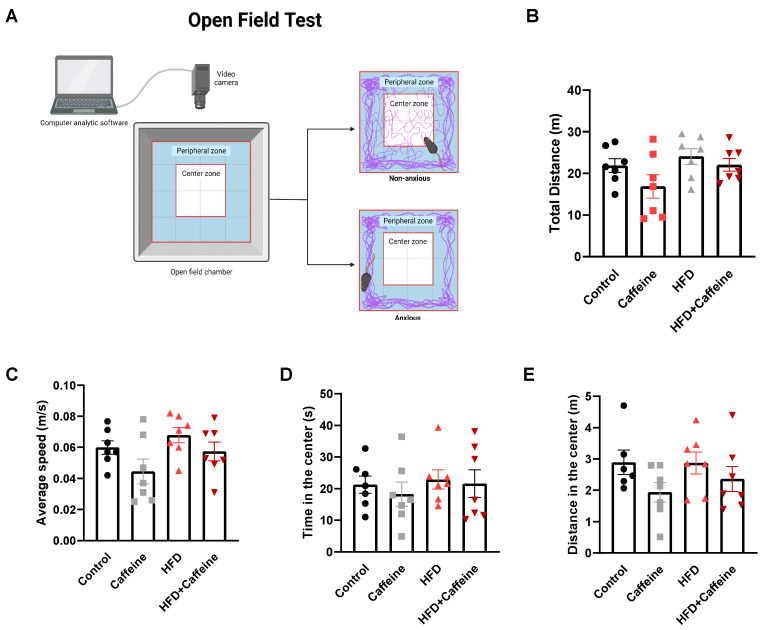
Effects of caffeine consumption on locomotor activity and anxiety-like behavior in the open-field test. (**A**) Schematic representation of the open-field test setup, illustrating the peripheral and center zones and the behavioral distinction between non-anxious (center exploration) and anxious (peripheral thigmotaxis) phenotypes. (**B**) Total distance travelled (m). (**C**) Average movement speed (m/s). (**D**) Time spent in the center zone (s). (**E**) Distance travelled in the center zone (m). Data are presented as individual values, mean ± SEM of *n* = 7 animals/group.

We used the open-field test to analyze the effects of caffeine consumption on locomotion and anxiety-like behavior. No differences were observed in locomotor activity (average speed, total distance) or anxiety-related parameters, including center entries and time spent in the center or periphery (Figure 4).

**Figure 5:**
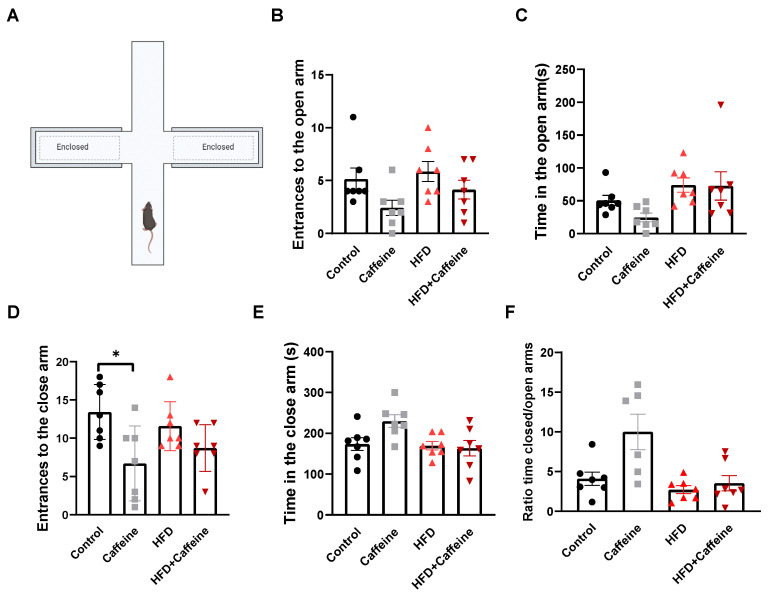
Effects of caffeine consumption on anxiety-like behavior in the elevated plus maze. (**A**) Schematic diagram of the elevated plus maze, consisting of two open arms and two enclosed arms. (**B**) Number of entrances into the open arms. (**C**) Time spent in the open arms (s). (**D**) Number of entrances into the closed arms. (**E**) Time spent in the closed arms (s). (**F**) Ratio of time spent in closed to open arms (anxiety index). Data are presented as individual values, mean ± SEM of *n* = 7 animals/group. * *p* < 0.05.

In the elevated plus maze, no differences were detected in open arm entries, time in open arms, or time in closed arms (Figure 5). The caffeine group showed fewer closed arm entries compared with controls (6.714 ± 2.007; *p* = 0.0107; Figure 5B). We also analyzed the ratio of time spent in closed/open arms, as a measure of anxiety-like behavior, and no changes were observed in any group.

### 3.4. Hypothalamic Neuropeptide Gene Expression

**Figure 6:**
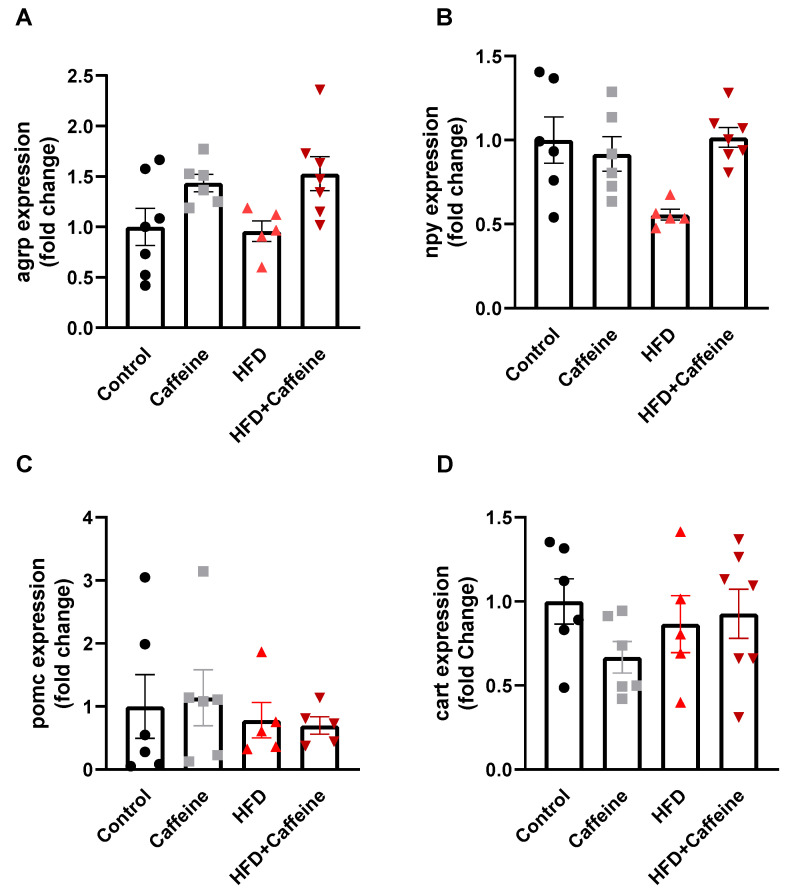
Effects of caffeine consumption on hypothalamic neuropeptide gene expression. Relative mRNA expression (fold change) of orexigenic and anorexigenic neuropeptides in the hypothalamus: (**A**) agrp (agouti-related peptide), (**B**) npy (neuropeptide Y), (**C**) pomc (pro-opiomelanocortin), and (**D**) cart (cocaine- and amphetamine-regulated transcript). Expression levels were normalized to control. Data are presented as individual values, mean ± SEM of *n* = 5–7.

We also analyzed the effect of chronic caffeine consumption in the expression of hypothalamic neuropeptides involved in the regulation of food intake. No differences were observed in Agrp, NPY, Pomc, and Cart expression (Figure 6A–D).

## 4. Discussion

The results of our study suggest that chronic caffeine consumption may have a protective effect against HFD-induced weight gain, adiposity, and improved glucose tolerance, independently of food intake. Importantly, our study showed that caffeine prevented not only weight gain but also adiposity gain, which is a great indicator of metabolic health, given that visceral adiposity itself, even without elevated body mass index (BMI), is associated with diabetes and cardiovascular disorders, as shown by a large recent cross-sectional study [35].

The effect of caffeine on glucose homeostasis appears to be dependent on whether it is administered acutely or chronically. In a randomized controlled trial (RCT), caffeine (200 mg oral, single dose) does not modify fasting glucose levels, but it decreases glucose tolerance when administered acutely to non-coffee drinkers. No effect was observed on insulin secretion during the GTT [36]. The authors hypothesized that this effect might be due to caffeine-induced catecholamines release, resulting in an acute hyperglycemic effect. However, consistent with our findings, chronic caffeine intake appears to have protective effects in glucose metabolism, as shown by important meta-analyses of observational studies, reporting an inverse relationship between coffee consumption and the risk of type 2 diabetes [37,38]. Similarly, chronic caffeine consumption improved glucose homeostasis, insulin/IGF-1 signaling, and reduced weight gain in diabetic pancreatomized rats [39]. Also, in HFD-fed mice, chronic caffeine (200 mg/L) consumption improved glucose tolerance and protected against insulin resistance, but it did not prevent weight or adiposity gain [40]. In this study, caffeine consumption lowered the levels of inflammatory adipocytokines, suggesting that the beneficial effects of caffeine might be attributable in part to the reduction in inflammation in the adipose tissue [40].

Comparable studies using 1 g/L caffeine in drinking water over 12 weeks reported prevention of weight gain and glucose tolerance, with additional reductions in triglycerides and cholesterol [41]. Differences in study outcomes may reflect variations in diet duration, caffeine dose, or fat content. Consistent with our findings, daily peripheral or central administration of caffeine has been reported to improve glucose tolerance, reduce adipocyte size, and prevent weight gain, effects mediated by A1R antagonism in the paraventricular nucleus (PVN). However, in this study, a decrease in food intake and blood triglycerides was reported following caffeine treatment, contrary to what we observed in our study, potentially reflecting differences in experimental duration (12 days vs. 8 weeks) or in dose and route of administration (60 mg/kg via gavage vs. ad libitum) [31].

In our study, caffeine-receiving groups exhibited increased food intake compared to controls, suggesting alternative mechanisms for its protective effects. The most plausible explanation is increased energy expenditure via brown adipose tissue (BAT) activation and thermogenesis. Acute IV or ICV caffeine administration induces neuronal activation in hypothalamic regions such as the lateral hypothalamus and dorsomedial hypothalamus, which drive BAT-mediated thermogenesis [42]. A low-dose crossover RCT (50 mg caffeine) also demonstrated increased thermogenesis without significant changes in energy intake or appetite, supporting this hypothesis [43]. However, BAT activation was not investigated in the present study.

The hypothalamic feeding circuit responds rapidly to food presentation and ingestion. Besides the increase in food intake observed in caffeine-treated groups, no changes were observed in the expression of hypothalamic neuropeptides involved in the regulation of food intake. Some studies have been shown that HFD consumption could disrupt feeding patterns, increasing daytime intake in mice [44,45,46]. However, in our study no changes were detected in total food intake in the HFD group, though we did not analyze the amount of food consumption in the day/night cycle. Adenosine released from tanycytes decreases AgRp and NPY expression in hypothalamic neuronal cell models, an effect abolished by A1R blockade [30]. As an adenosine receptor antagonist, caffeine is expected to exert opposite effects, contradictory to our data. The expression of the anorexigenic neuropeptide, POMC, was not changed in our study. However, this does not necessarily reflect unaltered anorexigenic signaling, since HFD consumption has been associated with increased POMC DNA methylation and dysfunctional protein expression, despite unchanged mRNA levels [47]. In a different study, POMC mRNA expression was decreased following HFD, suggesting that epigenetic mechanisms may also contribute [48]. Unfortunately, we did not investigate epigenetic mechanisms in our study.

Related to the behavior tests, our data showed that caffeine consumption does not induce an anxiety-like phenotype. However, neither HFD nor HFD + caffeine affected anxiety-like behavior in C57/BL6J mice. However, behavior results are variable between the studies depending on the experimental protocol [49]. Regarding the effect of caffeine consumption on the anxiety phenotype, chronic caffeine (0.3 g/L) intake for 2 weeks did not modify locomotor, mood or memory behavior [50]. On the contrary, it was demonstrated that acute administration of 100 mg/kg caffeine, but not at 25 or 200 mg/kg, reduces anxiety-like behavior [51]. Chronic caffeine consumption (1 g/L, 3 weeks) increased anxiety-like behavior in both the open-field and elevated plus maze tests, where the mice exhibited aggravated anxiety-like behavior, and these effects were abolished by global and hippocampal knockout of A2AR, highlighting the relevance of hippocampal A2A receptors in driving caffeine-induced behavioral changes [52]. In humans, a recent meta-analysis suggested that caffeine is associated with anxiety, with this relationship being stronger at higher doses or in sensitive individuals [49].

Under normal conditions, adenosine exerts a tonic inhibitory influence on dopamine signaling, dampening neuronal activity within reward-related circuits. By blocking A2A receptors, caffeine reduces this inhibitory tone and functionally enhances D2 receptor-mediated dopaminergic transmission [17]. This does not typically increase dopamine release directly but rather amplifies dopamine signaling efficiency, particularly in the mesolimbic and nigrostriatal pathways [53]. By blocking adenosine’s inhibitory effects, caffeine increases neuronal excitability and indirectly enhances dopamine transmission, especially within brain regions involved in motivation and reward. This dopaminergic modulation is often associated with improved alertness and increased psychomotor activity, but it can also contribute to heightened arousal, which in susceptible individuals may be interpreted as anxiety-like behavior. The overall effect of caffeine on anxiety therefore appears to be dose-dependent and context-dependent, with low to moderate doses generally producing stimulatory and potentially anxiolytic-like outcomes, whereas higher doses may exacerbate anxiety symptoms due to excessive central nervous system activation [54]. The dopaminergic system was also found to be altered in obesity by an increased brain expression of dopamine D2 receptors in important areas for energy homeostasis following DIO [55]. However, it was demonstrated that the availability of the dopamine D2 receptor was decreased in obese individuals in proportion to their BMI [56]. This is particularly important given that the D2 receptor appears to regulate plasma glucose levels and hepatic glucose metabolism through sympathetic innervation pathways [21]. Crucially, this central dopaminergic regulation extends beyond glucose homeostatic pathways to modulate peripheral aminotransferase dynamics. Recent insights into the dopamine–aminotransferase system suggest that central biogenic and trace amines act as key neurometabolic orchestrators, influencing hepatic AST and ALT activities to coordinate the metabolic shift between caloric catabolism and anabolism [22]. Caffeine was found to significantly reduce the levels of ALT and AST in the blood, as well as the overall level of fibrosis in the liver [57].

In rats, 9 weeks of HFD induced behavior impairments in the elevated plus maze test but not in the open field [58]. Using a 60% HFD, it was also reported that 4 weeks of HFD consumption induced behavior impairments in the elevated plus maze test in C57Bl6 mice [59]. As mentioned, part of the differences between our results and those listed above might be explained by the different fat percentages of HFD and time of HFD consumption, as well as the different animal models. Some studies have reported that elevated anxiety symptoms are linked to altered lipid profiles, including increased triglycerides and reduced HDL cholesterol in some studies [60,61]. It has been observed that there is a negative correlation between anxiety and high-density lipoprotein (HDL) levels, while higher triglyceride levels were observed in patients with depression and comorbid anxiety compared to depressive patients without anxiety [62].

In our study, we could find differences in lipid profile as well as anxiety parameters in the HFD mice model.

The absence of changes in locomotor activity in the open-field test indicates that the metabolic effects of caffeine observed here are unlikely to be driven by its classical psychostimulant properties. Although caffeine is widely recognized for increasing arousal and locomotion, these effects are dose- and context-dependent and may attenuate with chronic exposure [37]. Therefore, the reduction in body weight gain and adiposity observed in HFD-fed mice likely reflects metabolic adaptations, rather than increased physical activity per se. Behavioral assessments also showed that neither HFD nor caffeine affected anxiety-like behavior in C57BL/6J mice. Behavioral outcomes in HFD models vary across studies depending on diet duration, fat content, and experimental protocol [63,64].

## 5. Conclusions

In conclusion, chronic caffeine consumption counteracts key metabolic disturbances induced by a high-fat diet, including excessive weight gain, increased adiposity, and impaired glucose tolerance. These effects occur without reductions in food intake, indicating that caffeine’s protective actions are not mediated by anorectic effects. Behavioral analysis further suggests that these metabolic benefits are largely independent of changes in anxiety-like behavior or general psychostimulant effects. At the molecular level, neither high-fat diet exposure nor caffeine consumption altered hypothalamic neuropeptide expression. Overall, the data support a robust protective role of caffeine against diet-induced obesity and metabolic dysfunction. This protection is likely driven by mechanisms other than reduced energy intake, potentially involving increased energy expenditure and thermogenic processes. These findings highlight caffeine as a modulator of systemic energy balance and metabolic resilience under obesogenic conditions, warranting further investigation into its underlying mechanisms and translational relevance for metabolic disease prevention. This study has some limitations that should be considered when interpreting the findings. Only male C57BL/6J mice were used, which limits generalization, as sex differences in metabolism and response to caffeine are well known. Key mechanisms were not directly assessed, particularly energy expenditure, thermogenesis, and brown adipose tissue activation, so these remain speculative. In addition, hypothalamic analysis was limited to mRNA expression, without assessment of protein levels or neuronal activity due to a lack of samples. Finally, the translational relevance is limited, as the caffeine dose and route of administration (1 g/L in drinking water) may not reflect typical human consumption.

## Figures and Tables

**Table 1:** Mouse qRT-PCR primers sequences.

Gene	GenBankID
*POMC*	NM_008895
*AgRP*	NM_007427
*NPY*	NM_023456
*CART*	NM_013732
*GAPDH*	NM_008084

## Data Availability

The data presented in this study are available from the corresponding author upon reasonable request due to confidentiality restrictions and ongoing analyses for future studies.

## Abbreviations

The following abbreviations are used in this manuscript:

AgRP	Agouti-Related Peptide
AR	Adenosine Receptors
BAT	Brown Adipose Tissue
BMI	Body Mass Index
CART	Cocaine- and Amphetamine-Regulated Transcript
DALYs	Disability-Adjusted Life Years
GAPDH	Glyceraldehyde-3-phosphate dehydrogenase
GTT	Glucose Tolerance Test
HFD	High-Fat Diet
NPY	Neuropeptide Y
POMC	Pro-opiomelanocortin
PVN	Paraventricular Nucleus
